# Hydroxymethylbilane synthase (HMBS) gene-based endogenous internal control for avian species

**DOI:** 10.1186/s13568-020-01112-5

**Published:** 2020-10-07

**Authors:** Yaoyao Wang, Jilei Zhang, Kelly Patrick, Min Li, Jiansen Gong, Bu Xu, Qiuping Shen, Yi Yang, Lanjing Wei, Yuanyuan Zhang, Daxin Peng, Jianqiang Ye, Anil Poudel, Chengming Wang

**Affiliations:** 1 Yangzhou University College of Veterinary Medicine, Yangzhou, Jiangsu China; 2 Ross University School of Veterinary Medicine, Basseterre, West Indies St. Kitts and Nevis; 3 Poultry Institute, Chinese Academy of Agricultural Sciences, Yangzhou, Jiangsu China; 4 Department of Pathobiology, College of Veterinary Medicine, Auburn University, Auburn, Alabama USA

**Keywords:** Endogenous internal control, Birds, PCR, HMBS

## Abstract

With PCR becoming one of the most important and widely-used diagnostic tools for infectious diseases of poultry, an urgent need has developed for an endogenous internal control (EIC) that monitors the quality and quantity of poultry DNA in test samples. In this study we developed a SYBR-qPCR to target the poultry homolog of the hydroxymethylbilane synthase (HMBS) gene as an EIC for avian species. The avian HMBS-based qPCR was very sensitive, detecting one HMBS gene copy in a 20 µL reaction, and is highly specific for avian species. It amplified DNA from 11 organs and tissues of chickens showing it can be used as an EIC on a large variety of samples. The application of the established EIC on clinically and experimentally infected samples demonstrated that false negativity and result variations could result from samples being collected using different operators, techniques, preservatives, and storage times. The high sensitivity and specificity of the avian HMBS-based qPCR, its ability to quantify DNAs extracted from a wide range of tissues and poultry species along with its usefulness in reducing false negativity in PCR results associated with inadequate sampling and storage degradation makes it an ideal EIC for poultry DNA and RNA PCR diagnostics. The study also highlights the importance of appropriate sampling and storage of samples in ensuring accuracy of molecular diagnostic testing.

## Key points

- Established EIC (HMBS) is highly specific and sensitive.
- EIC was validated in samples from clinical and experimentally infected chickens.
- EIC reduced false negativity due to inadequate preservatives used and degradation during storage.

## Introduction

**Fig. 1:**
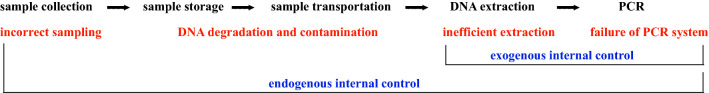
Exogenous and endogenous internal controls in molecular diagnostics. An exogenous IC is introduced into a test sample to monitor the efficiency of DNA extraction and PCR. In comparison, the endogenous internal control which is the reference genes of animal hosts and is present in the test sample can be used to monitor any potentials problems from sample collection and PCR during molecular diagnostics

Due to its exquisite sensitivity and specificity, polymerase chain reaction (PCR) has become one of the most valuable, powerful and widely-used tools for rapid detection of infectious agents in poultry (Okamatsu et al. 2016; Stoute et al. 2019; Hussein et al. 2019; de Wit et al. 2018; Kaltenboeck et al. 2005; Luan et al. 2016). However, the accuracy of PCR results is highly dependent on the quantity and quality of the DNA in the test sample. To monitor the efficiency of DNA extraction from samples, its concentration and degree of degradation, and the PCR operation itself, various exogenous and endogenous internal controls (IC) have been established (Fig. 1).

An exogenous IC usually consists of a defined copy number of target nucleic acids that is introduced into a test sample before extraction of DNA and RNA, and PCR. If the exogenous IC can be amplified by PCR as expected, it indicates that DNA has been extracted efficiently and the PCR system works properly (Rosenstraus et al. 1998; Bruggemann et al. 2000; Burggraf et al. 2004; Petersen et al. 2005; Huggett et al. 2005; Kalle et al. 2013). Usually, however, the exogenous IC is only applied to the test samples after they have been collected, stored and transported to a laboratory for processing. They thus do not evaluate these important phases of the process (Fig. 1). Endogenous internal controls (EIC) are not associated with these problems as they are reference genes of the host already present in the test sample. Any damage to the sample at any stage will interfere with the EIC and alert the operator that PCR results might be inaccurate and need further scrutiny.

Van Borm et al. (Van Borm et al. 2007) established a universal avian PCR EIC targeting the bird *β-actin* gene. As an extension of our previous work on establishing a mammalian EIC (Wei et al. 2014), we describe here the establishment and validation of a HMBS-based PCR as an EIC for poultry molecular diagnostics. Hydroxymethylbilane synthase (HMBS) is a protein which is an important enzyme in the heme biosynthetic pathway. The gene encoding the HMBS is a single-copy gene expressing HMBS in a wide variety of tissues in the mammalian body (Raich et al. 1989; Wang et al. 2012). We tested the sensitivity of the avian HMBS-based qPCR and its specificity as a poultry EIC using DNAs from a variety of bird and mammalian species. We also tested the suitability of the EIC for use with a variety of tissue and organ samples that can be collected, and the influence of sampling methods, different operators, and storage conditions on the performance of the EIC and how this correlates with diagnostic poultry PCRs.

## Materials and methods

### Avian HMBS-based qPCR

#### Primers and probes

Avian and mammalian sequences of the HMBS gene were obtained from GenBank to design specific primers for the poultry HMBS gene. The convenience sample of 11 bird species comprised: chicken, *Gallus gallus*, NC006111; turkey, *Meleagris gallopavo*, NC015036; pigeon, *Columba livia*, NW004973237; chimney swift, *Chaetura pelagica*; cuckoo, *Cuculus canorus*, NW009256549; bustard, *Chlamydotis macqueenii*, NW010464036; barn owls, *Tyto alba*, NW010024509; little egret, *Egretta garzetta*, NW009258617; fulmar, *Fulmarus glacialis*, NW009208351; Atlantic canary, *Serinus canaria*, NW007931173; and collared flycatcher, *Ficedula albicollis*, NC021695. The convenience sample of six mammalian species comprised: human, *Homo sapiens*, NG_000011; mouse, *Mus musculus*, NC_000075; dog, *Canis lupus familiaris*, NC006587; cat *Felis catus* NC_018732; cattle, *Bos taurus*, AC000172; and goat, *Capra hircus*, NC021695.

**Fig. 2:**
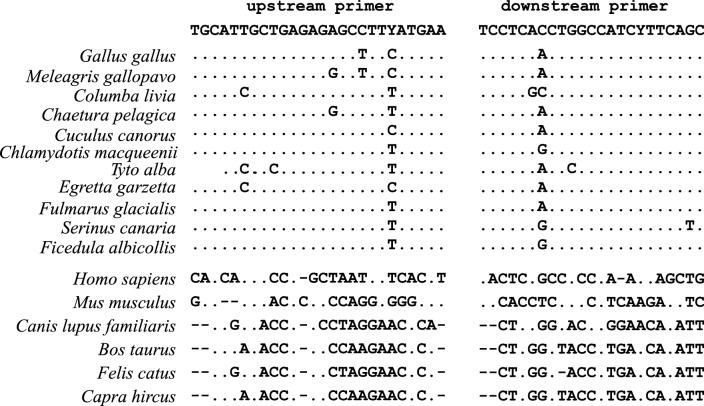
Alignment of the partial amplicons of the avian HMBS-based qPCR products for 11 bird species and 6 mammalian species. The nucleotide sequences of upstream and downstream primers in 11 bird species and 6 mammalian species are included in the boxes. The nucleotides between primers are not shown. Dots indicate that nucleotides are identical to the primers while dash denotes the deletion of the nucleotide. The upstream primer was used as shown while the downstream primer was used as the antisense oligonucleotide

The Clustal Multiple Alignment Algorithm was used to identify suitable primers in a highly conserved region of the HMBS gene of the bird species (Fig. 2) that would not detect mammalian HMBS gene. The primers to amplify a 340-bp target (Fig. 2) were synthesized by Integrated DNA Technologies (Coralville, IA, USA): upstream primer: 5′-TGCATTGCTGAGAGAGCCTTYATGAA-3′; downstream primer: 5′-GCTGAARGATGGCCAGGTGAGGA-3′.

#### Thermal cycling

The avian HMBS-based qPCR was performed in a LightCycler® 480II Real-time PCR platform (Roche) using a thermal protocol and PCR conditions as described (14) with a modified annealing temperature of 55 °C and 100 × SYBR Green 0.12 µL per 20 µL reaction, followed by high-resolution melting curve analysis.

#### Specificity

The specificity of the avian HMBS-based qPCR was verified using DNA from whole blood of seven chickens indigenous to China (Qingke chicken, Lanya Baitiao chicken, Luhua chicken, Rugao Huang chicken, Huxu chicken, Qingguan chicken, Baier chicken; kindly provide by the Poultry Institute of China), four rare bird species (wild goose, quail, swan, crane; kindly provided by the Yangzhou Zoo), a duck, goose, and pigeon, as well as DNAs from seven mammalian species (human, cat, dog, cattle, goat, sheep, and pig). PCR products were further verified using electrophoresis (1.5% MetaPhor agarose gels), followed by purification with a QIAquick PCR Purification Kit (Qiagen, Valencia, CA, USA), DNA sequencing (GenScript, Nanjing, Jiangsu, China) and BLASTn.

#### Sensitivity

For use as quantitative standards, the products of the avian HMBS-based qPCR on DNA extracted from the liver of a SPF AA broiler chicken were gel purified using a QIAquick Gel Extraction Kit (Qiagen, Valencia, CA, USA). To confirm the avian HMBS-based qPCR an aliquot of the products was sequenced at GenScript (Nanjing Jiangsu, China) and the remainder quantified (ng/ml) with the Quanti-iT ^TM^ PicoGreen ® dsDNA Assay Kit (Invitrogen Corporation, Carlsbad, CA, USA). After using the molecular mass of the HMBS gene to calculate the molarity of the solution, dilutions were made to give solutions containing 10^4^, 10^3^, 10^2^, 10^1^, 10^0^ gene copies per reaction. These were amplified with the avian HMBS-based qPCR in triplicate to determine the detection limit of the PCR.

### PCRs for AIV, ***Chlamydia*** and ***mcr-1***

Previously described and validated PCRs were used to detect avian influenza virus (AIV) (Luan et al. 2016), *Chlamydia* spp. (Guo et al. 2016) and the *mcr-1* gene (Zhang et al. 2017) in this study.

### Organs and tissues from chickens

The experiments in this study were reviewed and approved by the Institutional Animal Care and Use Committee of the Yangzhou University College of Veterinary Medicine. The student’s participation in this project to collect swabs was approved by the Institutional Review Board at Yangzhou University.

Four one-week-old healthy SPF AA broiler chickens were obtained from Sandeli Animal Husbandry Development Co., Ltd (Zhenjiang, Jiangsu, China). Following humane euthanasia, eleven organs and tissues were collected from each chicken and placed in Eppendorf tubes containing 800 µL DNA/RNA stabilization buffer (Roche Molecular Biochemicals, Indianapolis, IN) for DNA extraction as described below.

### Oropharyngeal swabs from chickens in live bird markets

Six student volunteers were trained so that each could identically carry out a protocol for collecting oropharyngeal swabs from chickens. They subsequently used this protocol to collect samples at a live bird market in their hometowns in six different provinces of China. The collected swabs were preserved in tubes containing an in-house DNA/RNA stabilization buffer for 7–10 days at room temperatures before being transported to Yangzhou University. The in-house DNA/RNA stabilization buffer contained 6M Guanidine-HCl, 20% (v/v) Triton X-100, 10 mM Urea and10 mM Tris-HCl (pH 4.4).

### Oropharyngeal swab collection from chickens infected with AIV and subsequent storage

#### Infection

Twenty SPF AA broiler chickens were individually tagged and housed in a level-two containment facility with free access to food and water. After three days, each chicken was inoculated intranasally with 100 µL of a suspension containing 10^6^ EID_50_ of avian influenza virus-H9N5.

#### Oropharyngeal swab collection

The oropharyngeal swabs were collected in three ways: (i) the thorough method: the swab was rotated in the throat 360° three times, and dragged along the tongue on removal; (ii) the intermediate method: the swab was rotated once in the throat 360°, and dragged along the tongue on removal; (iii) the gentle method: the swab was only dragged along the tongue, from posterior to anterior without making contact with the throat.

#### Influence of different oropharyngeal swab collection methods on the avian HMBS-based qPCR

Three days after the chickens were infected with the AIV, three swabs were taken from each chicken; one using the thorough method, one using the intermediate method and one using the gentle method. The swabs were placed in PBS and transported on ice to the laboratory where DNA extraction was performed within one hour of the swab collection.

#### Influence of storage media and duration on the avian HMBS-based qPCR and PCR for AIV

Four days following the AIV challenge, three oropharyngeal swabs were obtained using the thorough method and immediately frozen at -80 ºC until DNA/RNA extraction. Two hours later, a further three oropharyngeal swabs were obtained using the thorough method and placed in PBS, PBS and antibiotics, and the in-house DNA/RNA stabilization buffer. These were stored at room temperature for three days before DNA was extracted. After a further two hours, another three oropharyngeal swabs were obtained using the thorough method and placed in PBS, PBS with antibiotics, and the in-house DNA/RNA stabilization buffer. These were stored at room temperature for nine days before DNA was extracted.

### DNA extraction

Chicken tissues and organs were homogenized in a shaker (Bertin Technologies, France) with four 3.0 mm ceramic beads. The heart, liver, brain, spleen lung, adipose, kidney, and muscle samples were homogenized for two periods of 15 s (3160 g with a 15 s break in between) while the feather, bone and skin samples were homogenized for three periods of 20 s (310 g with 20 s breaks). DNA was extracted from the homogenates using the QIAgen® DNA Mini Kit (Qiagen, Valencia, CA, USA), eluted in 200 µL of 1× T_10_E_0.1_ buffer and stored at − 80 °C until assayed with the avian HMBS-based qPCR.

DNAs from reference strains of *Salmonella* Typhimurium (ATCC14028) and *Escherichia coli* (ATCC25922, ATCC8739) were extracted as described (Wei et al. 2014). Extracted DNAs of *Toxoplasma* strain RH and *Eimeria* sp. were kindly provided by the Laboratory of Veterinary Parasitology at the Yangzhou University College of Veterinary Medicine.

### Statistical analysis

Copies of the HMBS gene determined by the avian HMBS-based qPCR were log_10_-transformed and analyzed by means ± SD in factorial ANOVA. Comparisons of means under the assumption of no *a priori* hypothesis were performed by two-tailed Tukey honest significant difference (HSD) test. The chi-squared test was used to compare the positivity of HMBS, *Chlamydia*, AIV and *mcr-1* in different groups. Difference at P ≤ 0.05 was considered significant.

## Results

### Establishment of the avian HMBS-based qPCR

The BLASTn program showed that the sequences of the primers selected for the avian HMBS-based qPCR were highly specific and recognized all the bird species we intended to study (Fig. 2). They did not recognize other mammalian species, plants, fungi, bacteria, protozoa and arthropods. The avian HMBS-based qPCR was further verified when it amplified the DNAs from a duck, a goose, a pigeon, seven indigenous chicken species of China, and four rare bird species. It did not amplify DNAs from bacteria (*E. coli* and *Salmonella*), protozoa (*Toxoplasma* and *Eimeria*) and seven mammalian species (human, cat, dog, cattle, goat, sheep, and pig). The sensitivity of the avian HMBS-based qPCR was found to be a single copy of the target gene per 20 µL reaction.

### Presence of the HMBS gene in chicken tissues and organs

**Fig. 3:**
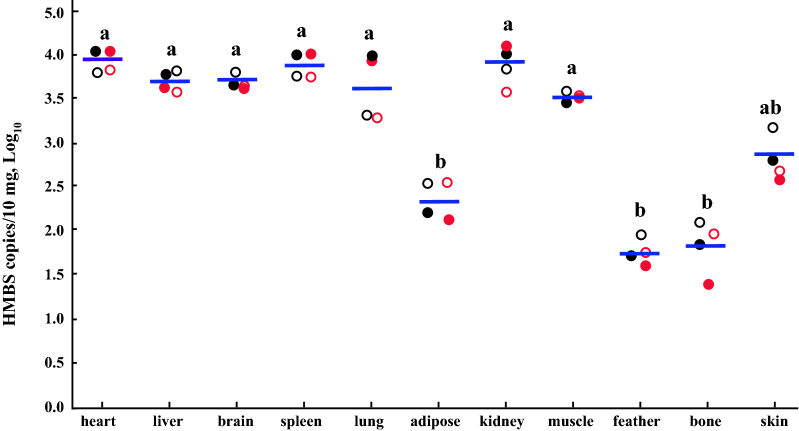
Distribution of avian HMBS-based qPCR copy numbers in chicken tissues and organs. The DNA of eleven organs/tissues from four chickens were tested in the avian HMBS-based qPCR to determine log10 transformed HMBS copies/10 mg. Blue bar denotes the mean value of the HMBS copies in the denoted organ or tissue. Different letters (a or b) indicate significant difference (P < 0.05)

The HMBS gene was found in all the organs and tissues of the chickens we studied but the HMBS copy numbers were significantly lower in adipose, feather and skin samples than in heart, liver, brain, spleen, lung, kidney and muscle samples (Fig. 3).

### Comparative evaluation of the molecular prevalence of the HMBS gene, AIV, *Chlamydia* and *mcr-1* in poultry oropharyngeal swabs

**Fig. 4:**
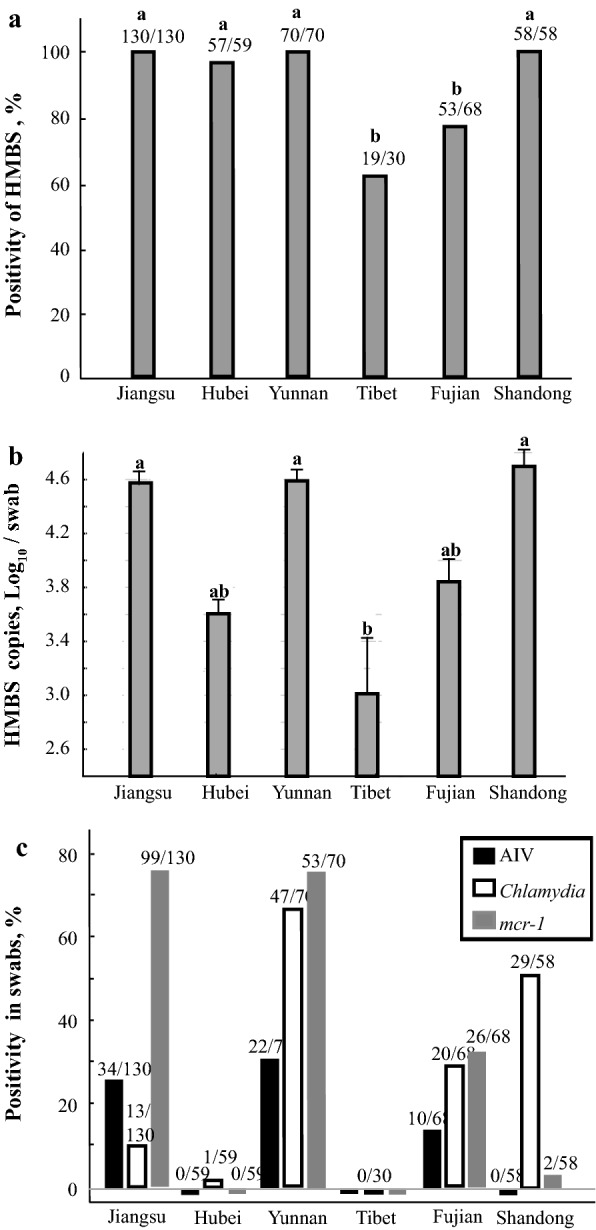
Molecular prevalence of HMBS, AIV, *Chlamydia* and *mcr-1* in poultry oropharyngeal swabs. Results of PCRs for HMBS, AIV, *Chlamydia* and *mcr-1* on oropharyngeal swabs collected by student volunteers from six provinces in China. The HMBS positivity from samples collected in Tibet and Fujian province was significantly lower than of those from four other provinces (**a**). In a similar trend, the HMBS copy numbers in swabs from Tibet were significantly lower than those of from Jiangsu, Yunnan and Shandong (**b**). Surprisingly, swabs from Tibet and Hubei with the lowest HMBS copy numbers showed lowest prevalences for AIV, *Chlamydia* and *mcr-1* by PCRs (**c**). The letter a or b indicates a significant

The HMBS gene, AIV, *Chlamydia* and *mcr-1* PCR results for the swabs collected by volunteer students in the different regions of China appeared to have a general pattern. Although the avian HMBS-based qPCR was positive for almost all the samples from the four provinces (93%; 387/415), there were significantly fewer positive swabs from Fujian (78%, 53/68) and Tibet (63.3%, 19/30) (Fig. 4a) as compared to the three provinces with 100% positive samples and one with 97% (Hubei). The HMBS copy numbers were also significantly lower in swabs from Fujian (10^3.9^ /swab) and Tibet (10^3.0^ /swab) when compared with those in swabs from the three other provinces (Jiangsu 10^4.6^ /swab; Yunnan 10^4.6^ /swab; Shandong 10^4.7^ Log_10_ /swab) (Fig. 4b). Samples from Hubei province also had comparatively low HMBS copy numbers (10^3.6^ /swab). Positive swabs for AIV, *Chlamydia* and *mcr-1* (Fig. 4c) varied considerably between provinces with Jiangsu and Yunnan having the most positive results (146 and 122, respectively) and Tibet the lowest (0). Shandong and Hubei also had low levels (31 and 1, respectively).

### Infection trial to evaluate the effect of sampling and storage buffer on PCR performance

**Fig. 5:**
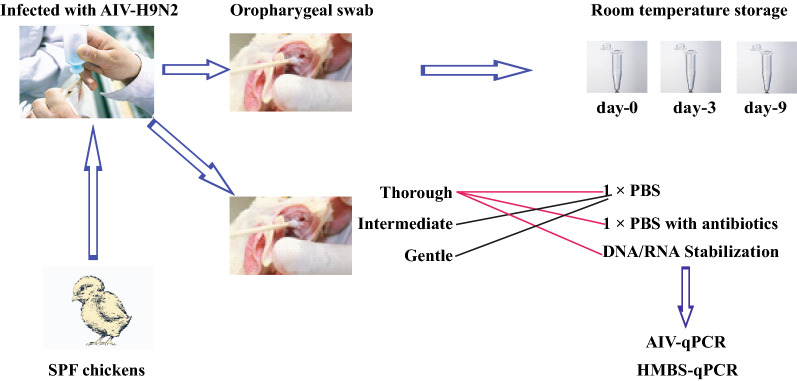
Infection trial to test the sampling methods and storage buffers. Twenty SPF chickens received intranasal inoculation of AIV-H9N2. Three-days post inoculation, oropharyngeal swabs were obtained from each chicken with gentle, intermediate and thorough swabbing into PBS for immediate DNA extraction. Thereafter, every two hours, the thorough swabbing was performed to obtain oropharyngeal swabs which were placed in PBS, in PBS with antibiotics, or in DNA/RNA stabilization buffer. The swabs in PBS were immediately frozen at − 80 °C until DNA extraction while those in PBS and antibiotics were stored for 3 days before freezing and those in the DNA/RNA buffer for 9 days before freezing before DNA extraction and HMBS and AIV PCRs

**Fig. 6:**
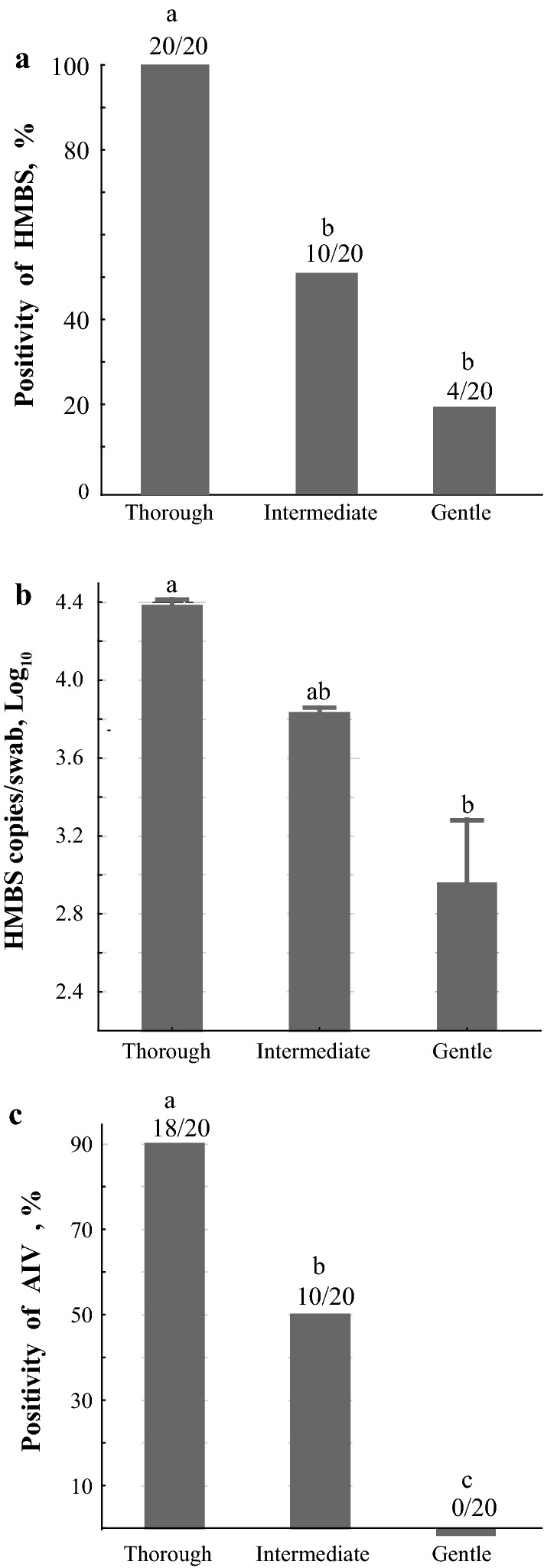
Influence of swabbing technique and HMBS and AIV copy numbers in experimentally infected chickens. Thorough swabbing resulted in the highest HMBS positivity and copy number, and the highest AIV positivity. In strong contrast, the swabs collected with gentle swabbing had the lowest HMBS positivity and copy number, with none being positive for AIV

All (100%; 20/20) the swabs collected using the thorough method from the chickens experimentally infected with AIV were positive in the avian HMBS-based qPCR and 90% (18/20) positive in the PCR for AIV (Fig. 5). The swabs collected using the thorough method also had the highest HMBS copy numbers (4.4 Log_10_ /swab) (Fig. 6). In strong contrast, the swabs collected with the gentle technique were least likely to be positive in the avian HMBS-based qPCR (20%; 4/20) and the PCR for AIV (0%; 0/20) and had the lowest HMBS copy numbers (10^3.0^ /swab) (Fig. 6). The swabs obtained with the intermediate technique gave intermediate values with each sample that became negative in the avian HMBS-based qPCR also becoming negative in the PCR for AIV.

**Fig. 7:**
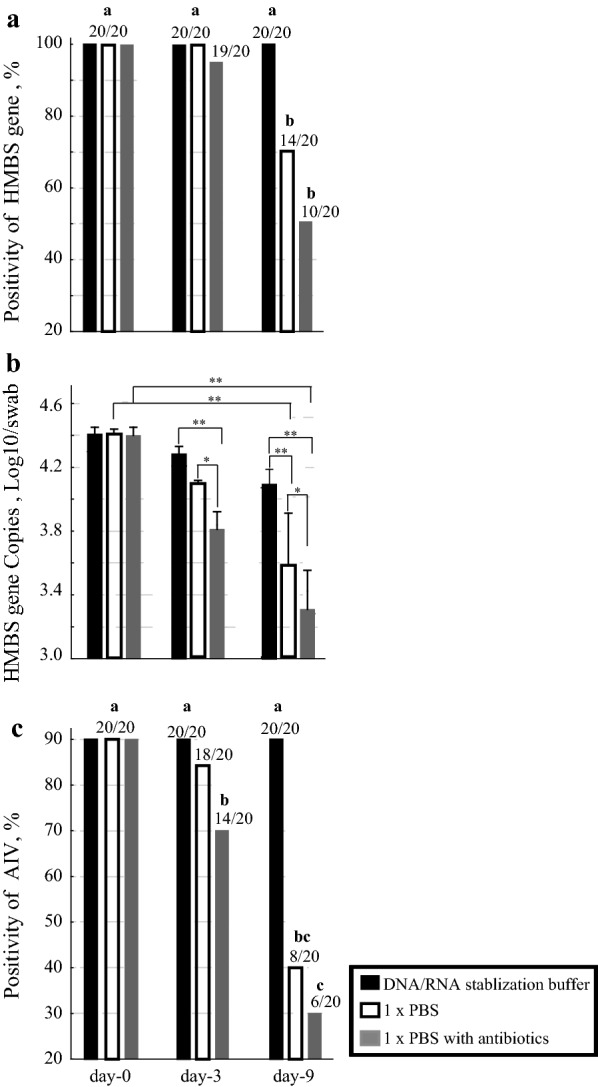
Effect of storage conditions of swabs collected using the thorough method on HMBS and AIV PCR results, and HMBS copy numbers. The percentages of HMBS (**a**) and AIV (**c**) PCR positive swabs stored in DNA/RNA stabilization buffer at room temperature remained the same after 3 (day-3) and 9 days (day-9). In contrast there was a decrease in the number of PCR positive swabs stored in PBS, and in PBS with antibiotics at room temperature over time. The HMBS copy numbers decreased over time in storage for all the swabs but the decrease was most marked in swabs stored in PBS with antibiotics (**b**)

With swabs collected identically but stored under different conditions before testing, we found no evidence of degradation of nucleic acids and decreased PCR performance in those that were stored in the DNA/RNA stabilization for 3 and 9 days at room temperature. All of these samples were positive (100%; 20/20) in the avian HMBS-based qPCRs and the PCRs for the AIV (Fig. 7). Swabs stored in PBS and particularly those stored in PBS with antibiotics, had substantially decreased positive results in the avian HMBS-based qPCR and AIV PCR after 3 and 9 days storage at room temperature (40%; 8/20 and 30%; 6/20, respectively). In all cases, the samples that became negative in the avian HMBS-based qPCR over time were also negative in the PCRs for AIV.

Although decreasing HMBS copy numbers were seen under all storage conditions, the decrease seen in swabs preserved in DNA/RNA buffer for 9 days (10^0.5^ =3.2 folds decrease in HMBS gene copy numbers) were significantly less for the swabs stored in PBS (10^0.8^=6.3 fold decrease), and those stored in PBS with antibiotics (10^1.2 ^= 15.8 fold decrease).

## Discussion

PCR has been widely used in the epidemiological investigation of various poultry pathogens and antibiotic resistance genes (Okamatsu et al. 2016; Stoute et al. 2019; Hussein et al. 2019; de Wit et al. 2018; Kaltenboeck et al. 2005; Luan et al. 2016; Zhang et al. 2017; Räty et al. 2005). To ensure the accuracy of PCR results, it is essential that appropriate and reliable EICs are available which can be used to monitor the entire process; sample collection, transport and storage, DNA extraction, and PCR. The SYBR-based PCR, detecting the avian HMBS gene that we designed and validated appears to fulfill the role of a universal poultry EIC. This PCR was very specific, detecting the target HMBS gene in a variety of avian species but not in other animals, bacteria and protozoa. The avian HMBS-based qPCR was positive on all 11 organs and tissues tested indicating it will be a useful EIC for PCRs conducted on a large variety of samples. It is of note that, probably because avian species have nucleated erythrocytes, the HMBS gene was most readily detected (highest gene copy numbers) in organs with high perfusion rates than in tissues with a lower blood supply such as feathers, skin and fat.

Although DNA concentrations of samples can be measured by absorbance at 260 nm (A_260_) and with fluorescent DNA-binding dyes, these methods indicate the total concentration of DNA including that in host tissue and contaminants, even if the DNA is degraded. In comparison, a PCR based EIC can be used to quantify only amplifiable and specific DNA of interest. In our avian HMBS-based qPCR we could detect as little as a single copy of the target HMBS gene in a 20 µL reaction volume. This high sensitivity is greater than that reported for other suggested EICs such as the bird β-actin gene which can only detect 10 to 1000 copies (Van Borm et al. 2007).

To evaluate the use of the avian HMBS-based qPCR as an EIC in a more clinical setting we investigated the effects of sample taking methods and subsequent sample storage on a PCR for AIV in chickens experimentally infected with the virus. We used AIV in our study as it is an RNA virus and RNA is more fragile than DNA and prone to degrade during preservation and storage (Bustin et al. 2004). In addition, the PCR for the AIV relies on reverse-transcription of the RNA in the virus into DNA and our EIC in this setting would thus be a monitor of both RNA and DNA quantity and stability.

The EIC findings using our avian HMBS-based qPCR indicated collecting samples into in-house RNA/DNA buffer effectively prevented DNA degradation over time, with all samples being positive at 0, 3, and 9 days post sampling. Consistent with the EIC results, the PCRs for AVI were also positive in all the experimentally infected chickens at 0, 3, and 9 days post sampling. In the case of samples stored in PBS and in PBS with antibiotics, however, the EIC indicated DNA degradation with fewer avian HMBS-based qPCR positive samples over time. This degradation of the DNA over time, indicated by the EIC, was reflected in the results of the PCRs for AIV which also had decreased numbers of positive results with increasing storage time and with samples stored in PBS and antibiotics. In all cases the samples that became negative in the avian HMBS-based qPCR over time were also negative in the PCRs for AIV indicating the close correlation between EIC results and those in diagnostic tests.

The usefulness of the avian HMBS-based qPCR as an EIC, even for RNA, was also evident in our studies investigating the effects of the methods used to obtain samples on the diagnostic PCR for AIV. The thorough swabbing method resulted in the EIC being positive with high copy numbers for all samples, of which 90% were also positive with the PCR for AIV. In samples from the same chickens collected using the moderate method, however, the EIC indicated the samples collected in this way were not adequate with only 50% (10/20) now being positive in the avian HMBS-based qPCR. These 10 samples were all positive in the PCR for AVI. In samples collected with the gentle method, only 20% (4/20) were positive with the EIC which was reflected in the low levels of samples positive in PCRs for AIV (0%; 0/20). Again, in all cases the samples that were negative in the avian HMBS-based qPCR used as an EIC were also negative in the PCRs for AIV further confirming the usefulness of this EIC.

The importance of the operator in obtaining adequate samples was demonstrated by the results we obtained with student volunteers. Despite receiving identical training in the required swabbing technique there were considerable differences between samples taken by different operators in the EIC results we obtained with our avian HMBS-based qPCR. Although we cannot exclude the possibility that there is considerable variation in the prevalences of AVI, *Chlamydia* and *mcr-1* in the different provinces, we suspect that at least some of the negative data was due to sample errors. Without the EIC data the possibility of operator inconsistency might have escaped our attention.

With all molecular diagnostic testing it is essential to prevent DNA and RNA degradation during transport and storage before nucleic acid extraction and PCR. Our data clearly demonstrates the advantages of DNA/RNA stabilization buffer in preserving DNA and RNA integrity. The guanidine contained in the RNA/DNA stabilization buffer is a strong protein denaturant and detergent which causes complete and immediate cell lysis and inactivation of nucleases. It also kills any microbes in the samples, thereby preventing them from releasing nucleases into the sample. The end result is stabilization of the nucleic acids in test samples which enables long-term storage and transport to laboratories for processing without increasing the risk of false negative results. The AIV infection model we studied shows clearly that copy numbers in positive PCRs decrease significantly with time and false negative results increase significantly with time when samples are stored in PBS, and PBS and antibiotics.

In conclusion, the highly sensitive and specific HMBS-based RT-PCR we established and validated in our experiments appears to be useful as an EIC for both DNA and RNA based avian diagnostic PCRs. The AIV infection trial we carried out highlights the importance of correct sampling and proper storage and transport of samples to ensure the accuracy of PCRs. Guanidine-containing DNA/RNA stabilization buffer proved highly effective in preserving quality DNA and RNA in our studies.

## Data Availability

All data related to this work are included in this manuscript.

## References

[CR1] Bruggemann J, Stephen J, Chang YJ, Macnaughton S, Kowalchuk G, Kline E, White DC (2000). Competitive PCR-DGGE analysis of bacterial mixtures an internal standard and an appraisal of template enumeration accuracy. J Microbiol Methods.

[CR2] Burggraf S, Olgemöller B (2004). Simple technique for internal control of real-time amplification assays. Clin Chem.

[CR3] Bustin SA (2004). Nolan T (2004) Pitfalls of quantitative real-time reverse-transcription polymerase chain reaction. J Biomol Tech..

[CR4] de Wit JJ, Cazaban C, Dijkman R, Ramon G, Gardin Y (2018). Detection of different genotypes of infectious bronchitis virus and of infectious bursal disease virus in European broilers during an epidemiological study in 2013 and the consequences for the diagnostic approach. Avian Pathol.

[CR5] Guo W, Li J, Kaltenboeck B, Gong J, Fan W, Wang C (2016). Chlamydia gallinacea, not *C. psittaci*, is the endemic chlamydial species in chicken (*Gallus gallus*). Sci Rep..

[CR6] Huggett J, Dheda K, Bustin S, Zumla A (2005). Real-time RT-PCR normalisation: strategies and considerations. Genes Immunol.

[CR7] Hussein EA, Hair-Bejo M, Liew PS (2019). Infectious bursal disease virus tissue tropism and pathogenesis of the infection in chickens by application of in situ PCR, immunoperoxase and HE staining. Microb Pathog.

[CR8] Kalle E, Gulevich A, Rensing C (2013). External and semi-internal controls for PCR amplification of homologous sequences in mixed templates. J Microbiol Methods.

[CR9] Kaltenboeck B, Wang C (2005). Advances in real-time PCR: application to clinical laboratory diagnostics. Adv Clin Chem.

[CR10] Luan L, Sun Z, Kaltenboeck B, Huang K, Li M, Xu X, Ye J, Li J, Guo W, Wang C (2016). Detection of influenza A virus from live-bird market poultry swab samples in China by a pan-IAV, one-step reverse-transcription FRET-PCR. Sci Rep.

[CR11] Okamatsu M, Hiono T, Kida H, Sakoda Y (2016). Recent developments in the diagnosis of avian influenza. Vet J.

[CR12] Petersen D, Dahllöf I (2005). Improvements for comparative analysis of changes in diversity of microbial communities using internal standards in PCR-DGGE. FEMS Microbiol Ecol.

[CR13] Raich N, Mignotte V, Dubart A, Beaupain D, Leboulch P, Romana M, Chabret C, Charnay P, Papayannopoulou T, Goossens M, Romeo PH (1989). Regulated expression of the overlapping ubiquitous and erythroid transcription units of the human porphobilinogen deaminase (PBG-D) gene introduced into non-erythroid and erythroid cells. J Biol Chem.

[CR14] Räty R, Rönkkö E, Kleemola M (2005). Sample type is crucial to the diagnosis of *Mycoplasma pneumoniae* pneumonia by PCR. J Med Microbiol.

[CR15] Rosenstraus M, Wang Z, Chang SY, DeBonville D, Spadoro JP (1998). An internal control for routine diagnostic PCR: design, properties, and effect on clinical performance. J Clin Microbiol.

[CR16] Stoute ST, Jackwood DJ, Crossley BM, Michel LO, Blakey JR (2019). Molecular epidemiology of endemic and very virulent infectious bursal disease virus genogroups in backyard chickens in California, 2009–2017. J Vet Diagn Invest.

[CR17] Van Borm S, Steensels M, Ferreira HL, Boschmans M, De Vriese J, Lambrecht B, Berg T (2007). A universal avian endogenous real-time reverse transcriptase-polymerase chain reaction control and its application to avian influenza diagnosis and quantification. Avian Dis.

[CR18] Wang C, Mount J, Butler J, Gao D, Jung E, Blagburn BL, Kaltenboeck B (2012). Real-time PCR of the mammalian hydroxymethylbilane synthase (HMBS) gene for analysis of flea (Ctenocephalides felis) feeding patterns on dogs. Parasitol Vectors.

[CR19] Wei L, Kelly P, Zhang J, Yang Y, Zheng X, Tao J, Zhang Z (2014). Wang C (2014) Use of a universal hydroxymethylbilane synthase (HMBS)-based PCR as an endogenous internal control and to enable typing of mammalian DNAs. Appl Microbiol Biotechnol..

[CR20] Zhang J, Wang J, Chen L, Yassin AK, Kelly P, Butaye P, Li J, Gong J, Cattley R, Qi K, Wang C (2017). Housefly (Musca domestica) and Blow Fly (*Protophormia terraenovae*) as vectors of bacteria carrying colistin resistance genes. Appl Environ Microbiol.

